# Malignant Rhabdoid Tumor, an Aggressive Tumor Often Misclassified as Small Cell Variant of Hepatoblastoma

**DOI:** 10.3390/cancers11121992

**Published:** 2019-12-11

**Authors:** Ladan Fazlollahi, Susan J. Hsiao, Manpreet Kochhar, Mahesh M. Mansukhani, Darrell J. Yamashiro, Helen E. Remotti

**Affiliations:** 1 Department of Pathology and Cell Biology, Columbia University Irving Medical Center, New York Presbyterian Hospital, New York, NY 10032, USA; sjh2155@cumc.columbia.edu (S.J.H.); mm322@cumc.columbia.edu (M.M.M.); dy39@cumc.columbia.edu (D.J.Y.); her2007@cumc.columbia.edu (H.E.R.); 2 Department of Pediatrics, Division of Pediatric Hematology, Oncology, Stem Cell Transplantation, Columbia University Irving Medical Center; New York, NY 10032, USA; mkk9008@nyp.org

**Keywords:** Malignant Rhabdoid Tumor (MRT), Hepatoblastoma, small cell Hepatoblastoma, small cell undifferentiated (SCUD) Hepatoblastoma, SMARCB1, INI1

## Abstract

The clinical management of pediatric liver tumors involves stratification into risk groups. One previously defined, high-risk group of hepatoblastomas is the small cell undifferentiated variant. In light of molecular studies showing *SMARCB1* deletion in these tumors, it is now recognized that most small cell, undifferentiated liver tumors represent an aggressive unrelated tumor—the malignant rhabdoid tumor (MRT). *SMARCB1* is a member of the chromatin remodeling SWI/SNF complex and encodes the INI1 protein. The histologic diagnosis of MRT is currently based on INI1 negative immunoreactivity and the presence of rhabdoid morphology. INI1-negative small cell liver tumors lacking classic rhabdoid morphology are often misclassified as small cell undifferentiated hepatoblastomas (SCUD-HB), according to the current classification. Pediatric liver tumors diagnosed between 2003–2017 as SCUD-HB (four cases) or MRT (two cases) were identified from the Columbia University Pathology Department Archives. All tumors were associated with normal or low serum alpha fetoprotein levels, and showed an absence of immunohistochemical staining of hepatocellular markers (Hep-par1, Arginase) and loss of INI1 staining. Two cases were initially diagnosed as MRT, one with prominent rhabdoid morphology, the other with predominant small cell morphology. The remaining four cases with small cell morphology were classified as SCUD-HB. Ancillary molecular studies confirmed the loss of *SMARCB1*, supporting the diagnosis of MRT in all cases, proving morphology an unreliable criterion. It is critical to eliminate the term INI1-negative hepatoblastoma from the current classification scheme, and classify INI1-negative tumors as MRT, particularly since high-risk HB-chemotherapy regimens are not effective for treating MRT.

## 1. Introduction

Rhabdoid tumors are highly aggressive tumors of infancy and early childhood. They have been reported in multiple sites, including the central nervous system (known as atypical teratoid/rhabdoid tumors) [1,2,3], kidneys (referred to as rhabdoid tumors of the kidney), and soft tissue (malignant rhabdoid tumors) [4,5]. The liver as a primary site of malignant rhabdoid tumors was first described by Gonzalez-Crussi in 1982 [6]. Malignant rhabdoid tumors (MRT) of the liver are a distinct group of tumors of unknown cell of origin with aggressive clinical behavior, refractory to chemotherapy and radiation, and with high mortality [7,8,9]. MRT of the liver are uncommon, comprising less than 5% of malignant liver tumors in infants [7,10,11].

Historically MRT was morphologically identified as a unique tumor type based on its characteristic “rhabdoid” cytologic features with abundant eosinophilic cytoplasm and eccentric nuclei [6]. In the era of molecular genomics, regardless of their anatomical site, the majority of MRT show loss of the protein product of the *SMARCB1* gene, also known as BAF47/INI1/hSNF5 [12,13]. The *SMARCB1* gene located on chromosome 22q11.2 is a core subunit of the ATP-dependent chromatin remodeling SWItch/Sucrose Non-Fermentable (SWI/SNF) complex [14,15,16,17]. The SWI/SNF complex controls gene transcription [18] and has a tumor suppressing function [19]. Studies have shown that biallelic inactivation of *SMARCB1* seem to be both necessary and sufficient to cause cancer [11,16,20]. All rhabdoid tumors with homozygous mutations and/or deletions of *SMARCB1* show loss of nuclear expression of INI1/BAF47 protein, that can be detected by immunohistochemistry [21,22].

In the current classification of pediatric liver tumors, INI1-negative immunostaining in the absence of rhabdoid morphology is insufficient to diagnose MRT of the liver. Therefore, based on the current guidelines, MRT cases lacking classic rhabdoid morphology are often misdiagnosed as SCUD-HB, if not tested for *SMARCB1* deletion [23]. According to the most current Children’s Oncology Group (COG), the classification [24] and College of American Pathologists (CAP) guidelines [25] small cell undifferentiated hepatoblastoma (SCUD-HB) is a subtype of epithelial hepatoblastoma with adverse outcome [21] that can have variable INI1 immunoreactivity. Recent studies have shown that adverse clinical outcomes occur in small cell HB INI1 negative cases [9,26] whereas no worse outcome is noted in small cell HB INI1 positive cases [27]. In this study, we retrospectively examined all cases at our institution diagnosed as small cell HB and MRT, in order to characterize the similarities and differences between these two tumors, examining clinical presentation, clinical outcome, and morphologic, immunophenotypic and molecular characterization.

## 2. Materials and Methods

### 2.1. Patient Samples

After institutional review board approval was obtained (Protocol Number: IRB-AAAM9156), a retrospective search for the pediatric liver tumors diagnosed as small cell undifferentiated hepatoblastoma (SCUD-HB) or malignant rhabdoid tumor (MRT) was performed in patients diagnosed between 2000 and 2017 in the database archive of Columbia University Department of Pathology. A total of six cases were identified. Two separate pathologists reviewed all cases. 

### 2.2. Immunohistochemistry

Immunohistochemical staining was performed on 5-micron cut sections of formalin-fixed, paraffin-embedded (FFPE) tissue blocks of all cases on Ventana staining system (Ventana Medical Systems, Tucson AZ, USA). All cases were stained with INI1 (monoclonal mouse antibody; 1:400; BD Bioscience, San Jose, CA, USA), Hep-par1 (mouse monoclonal antibody; 1:200; Dako, Santa Clara, CA, USA), Arginase (rabbit monoclonal antibody; 1:100; Sigma-Aldrich, St. Louis, MO, USA) and glypican-3 (mouse monoclonal antibody; ready to use; Sigma-Aldrich, St. Louis, MO, USA) antibodies. 

### 2.3. Molecular Analysis

#### 2.3.1. Somatic Copy Number Variant Analysis (SCNA)

Sequencing of tumor samples was performed using the Columbia Combined Cancer Panel (CCCP), as previously described [28]. In brief, 50–200 ng DNA was sheared with a Covaris S2 Sonication system and targeted sequences of 467 genes were captured using Agilent SureSelect capture reagents (Santa Clara, CA, USA). Sequencing was performed on Illumina HiSeq 2500 at 2 × 100 bp paired-end reads. For SCNA detection by CCCP, the fragments per kilobase of exon per million mapped reads (FPKM) was calculated by NextGENe software (version 2.3.4, Softgenetics, State College, PA, USA). The weighted average was determined and compared to average values, obtained from either 18 female normal control samples or 14 male normal control samples, to determine the fold change. The number of copies (n) was inferred from the fold change (FC) based on the tumor purity (P) for each sample, (*n* = [(200 × FC) − 2 × (100 − P)]/P). 

#### 2.3.2. Cancer Whole Exome Sequencing and Transcriptome

Cancer whole exome and transcriptome sequencing was performed on two cases, as previously described [29]. In brief, 250 ng of genomic DNA was sheared using a Covaris S2 Sonication system (Woburn, MA, USA) and exonic sequences were captured using Agilent SureSelectXTAll Exon V5 + UTRs reagents. Sequencing was performed on the Illumina HiSeq2500, using paired-end 100 cycle × 2 sequencing. Mapping, variant calling of tumor and normal samples was performed using NextGENe software and annotation, and filtering was performed using an in-house developed pipeline. Copy number changes were identified using EXCAVATOR software (v.2.2; https://sourceforge.net/projects/excavatortool [30]). RNA was sequenced using the TruSeq Stranded Total RNA LT Sample Prep Kit on the Illumina HiSeq2500 (San Diego, CA, USA). Data were analyzed using the Tuxedo Suite Package. Unmapped reads were analyzed using FusionMap (01_2018 version ) to identify translocations and relative expression levels were determined relative to an in-house database of control samples.

## 3. Results

### 3.1. Clinical and Histopathologic Characterization of MRT

**Figure 1:**
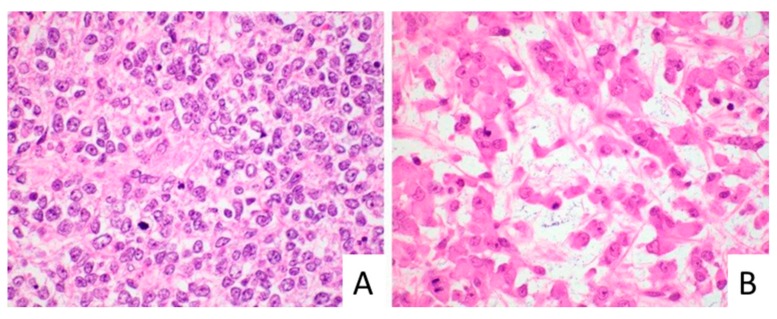
Malignant rhabdoid tumor (MRT) showing small cell morphology (**A**, case 1) versus typical rhabdoid morphology (**B**, case 3), original magnification 600×.

We identified six cases of pediatric liver tumors in our Intradepartmental Archives diagnosed as SCUD-HB or MRT between 2003 and 2017. Four of the six cases had a diagnosis of SCUD-HB and two cases had a diagnosis of MRT at the time of initial diagnosis. The patient age at the time of diagnosis ranged from 3 to 24 months (median age of 10.5 months). All six patients had low or normal serum levels of alpha-fetoprotein (AFP), AFP < 100.0 ng/mL (range 3.4 to 90.0 ng/mL) with (median AFP level of 24 ng/mL). Abdominal computed tomography (CT) with contrast showed a heterogeneous parenchymal liver mass in all patients. All patients had an initial percutaneous diagnostic liver biopsy. In four patients, the post-treatment liver resection (partial hepatectomy) was also available. In four cases (1, 2, 4 and 5, Table 1), the biopsy showed pure small cell morphology, with nests and sheets of small tumor cells with round to ovoid nuclei and scant cytoplasm. These cases were initially diagnosed as small cell undifferentiated hepatoblastoma. In the remaining two cases, one (3) had areas with larger tumor cells with prominent nucleoli and eosinophilic inclusions, compatible with rhabdoid morphology. This case was diagnosed as MRT on the initial diagnostic biopsy. The other case (6) had predominantly small cell morphology, but the patient presented in 2017 and, on the basis of loss of INI1 on immunohistochemistry, was diagnosed as MRT on the initial biopsy. Figure 1 illustrates the different morphologic features of small cell morphology and rhabdoid morphology. 

### 3.2. Immunohistochemical Profile of MRT

**Figure 2:**
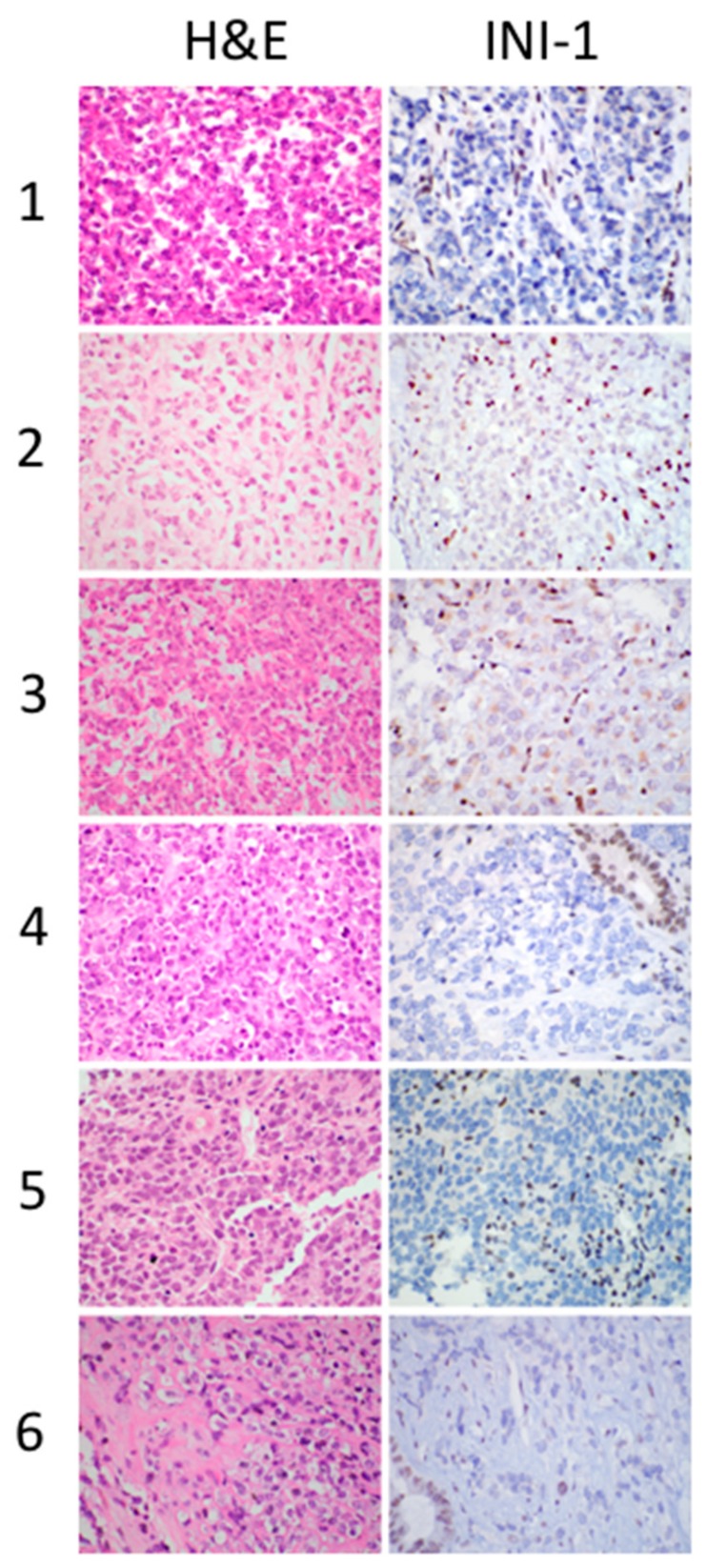
MRT cases (1–6), showing the spectrum of morphologic features, with predominant small cell morphology. All cases were INI1 negative by immunohistochemistry, original magnification 600×.

Immunohistochemical staining for INI1 showed a loss of INI1 protein in tumor cells with positive internal control (endothelial and inflammatory cells with retained nuclear INI1) in all six cases (Figure 2). The tumor cells were negative for hepatocellular differentiation markers (Hep-par1 and Arginase), however, the immunostains highlighted the entrapped non-neoplastic hepatocytes within the tumor. Among the five cases with small cell morphology, three presented before 2015, and therefore no confirmatory molecular testing was performed at the time of diagnosis. These cases were classified as small cell undifferentiated hepatoblastoma (SCUD-HB) based on their morphology and he absence of rhabdoid features, following the diagnostic guidelines of classification at the time of diagnosis. 

### 3.3. Molecular Characterization of MRT

**Figure 3:**
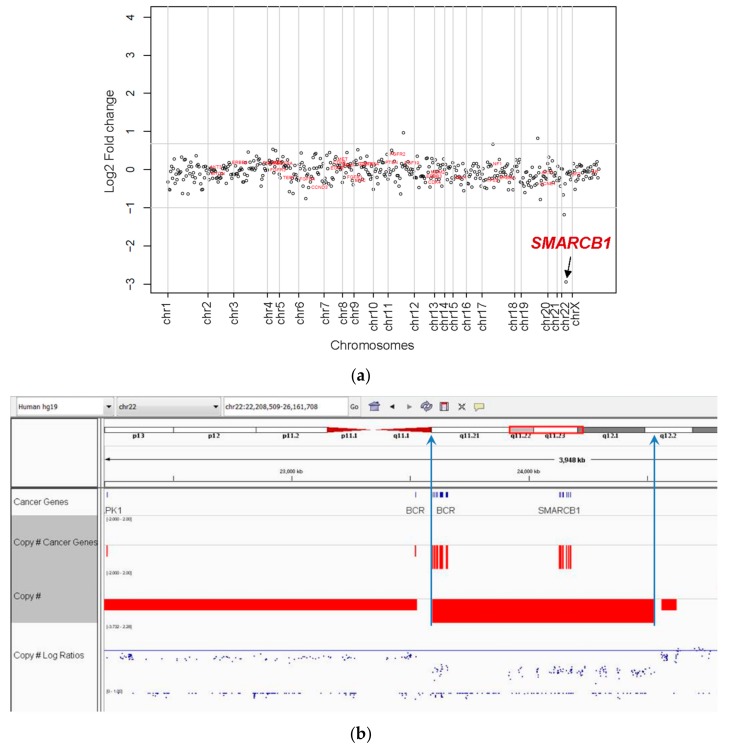
(**a**) Copy number analysis by CCCP. The log2-fold change for each gene is shown. SMARCB1 (indicated by arrow) on chromosome 22 shows a fold change consistent with biallelic deletion. (**b**) Cancer whole exome sequencing (CWES) was performed in two cases and confirmed bi-allelic *SMARCB1* gene deletion involving chromosome 22q11.2.

Columbia Combined Cancer Panel (CCCP) analysis was performed on six cases, and cancer whole-exome and transcriptome analysis were performed on two cases. CCCP copy number analysis showed loss of *SMARCB1* on chromosome 22q11.2 in all six cases. Copy number analysis for a representative case is shown in Figure 3a. When adjusted for tumor purity, the estimated number of copies of *SMARCB1* for each case was zero copies, consistent with the biallelic deletion of *SMARCB1* in all six tumors. CCCP copy number analysis of non-tumor tissue was performed in five of six cases, with all cases tested (five of five) showing no evidence of germline deletion of *SMARCB1* in non-neoplastic liver tissue. CWES and transcriptome sequencing was also performed in two cases (#4,6) and confirmed biallelic deletion of *SMARCB1* (Figure 3b). For Patient 4, CWES findings demonstrating the biallelic deletion of *SMARCB1* were described in a prior published case report [31]. 

**Figure 4:**
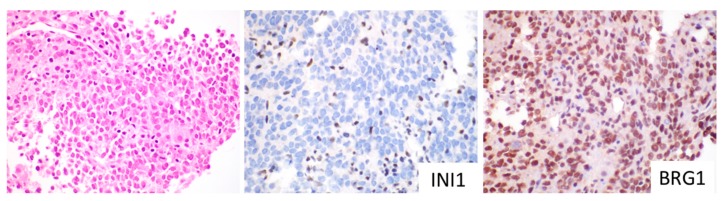
MRT (Case 5) with molecular findings of *SMARCB1* biallelic deletion and *SMARCA4* VUS. Immunostaining showed the absence of INI1 and presence of BRG1, original magnification 600×.

One *SMARCA4* variant of uncertain significance (VUS) was identified. In this case, IHC evaluation showed preserved BRG1 protein in the tumor (Figure 4). Pathogenic *SMARCA4* variants typically show an absent BRG1 protein by IHC, therefore, the presence of an intact BRG1 protein in this case supported the designation of *SMARCA4* VUS. In addition, INI1 was negative by IHC in this case (Figure 4). To our knowledge, no MRT cases published to date have shown concomitant mutations/deletions of *SMARCA4* and *SMARCB1*. In this case, expression analysis showed INI1 expression to be in the <1^st^ percentile (relative to control samples), which is consistent with the negative INI1 immunohistochemical staining and *SMARCB1* biallelic deletion seen. The clinical, morphologic and molecular profile of the cases are summarized in Table 1. 

### 3.4. Clinical Treatment and Outcome of MRT

At initial clinical presentation three patients had Stage II disease (1, 5, 6), and three patients had Stage IV disease with lung metastases (2, 3, 4). All patients received chemotherapy. Patient 1 had an initial resection with tumor rupture at time of surgery, followed by chemotherapy. Three patients after induction chemotherapy underwent tumor resection, followed by radiotherapy and post-operative chemotherapy. Two patients (2, 5) developed progressive disease following the initial chemotherapy and tumor resection was not performed. One of these patients was started on a phase-1 trial using pazopanib monotherapy, but was only able to tolerate the medication for two weeks due to disease progression and ultimately was removed from the trial. Four patients died of disease. These four patients were initially diagnosed as SCUD-HB. The other two patients diagnosed with MRT on the initial biopsy are alive 18 months and eight years from diagnosis. One of these patients (3) was found to have pulmonary metastases at diagnosis, the largest measuring up to 13 × 6 mm. All lung nodules completely resolved with the administration of pre-operative chemotherapy, in the span of two months (five cycles). The treatments and patient outcomes are summarized in Table 2. 

## 4. Discussion

### 4.1. Establishing the Diagnosis of Hepatic MRT

#### 4.1.1. MRT Are Tumors Typically Characterized by SMARCB1 Deletions with INI1 Loss

MRT of the liver represent a distinct pediatric malignancy driven by the homozygous loss of *SMARCB1* [32]. Although molecular genetic studies of rhabdoid tumors typically reveal the biallelic inactivation of the *SMARCB1* gene, other tumor types (schwannomas, meningiomas) may also show deletions or mutations of the *SMARCB1* gene that lead to a loss of INI1 protein expression. Since loss of INI1 does not exclusively occur in MRT, the aggressive small cell variant of HB was initially thought to represent an INI1-deficient tumor, distinct from MRT [21]. Another point to consider in the diagnosis of MRT is that a small percent of MRT show retention of the INI1 protein, and have mutations in a second locus of the SWI/SNF complex, the *SMARCA4* gene, resulting in the absence of the BRG1 protein [17,33,34,35,36]. For practical diagnostic utility, INI1-negative liver tumors in the appropriate clinical context (young children with liver tumors with small cell or rhabdoid features with low AFP levels) represent MRT. If liver tumors show rhabdoid or small cell features and express INI1, these tumors should then be evaluated for BRG1 protein, to exclude the rare MRT associated with *SMARCA4* inactivation.

#### 4.1.2. MRT and SCUD-HB Represent the Same Tumor

It has becoming increasingly clear that the two historically separate groups of primary pediatric liver tumors showing characteristic aggressive clinical behavior include HB with small cell undifferentiated morphology and MRT with rhabdoid morphology that represent the same INI1 deficient tumor. As in the case of rare tumors, appreciation of the spectrum of histologic features characterizing the tumor increases over time. Rhabdoid tumors of the kidney and brain have also been misclassified as small cell variants of more common tumors of these sites. In the brain, it has been increasingly recognized that rhabdoid tumors often do not show characteristic rhabdoid morphology and often are composed of small cells with relatively scant cytoplasm [3,37]. Historically, AT/RT of the brain with small cell morphology were misclassified as a variety of tumors, most commonly variants of medulloblastomas [37]. In the brain, INI1 negative immunoreactivity in conjunction with small cell morphology can establish the diagnosis of MRT. 

### 4.2. MRT of the Liver Most Commonly Have a Small Cell Morphology

MRT of the liver often have predominant small cell morphology, occur in very young children, have low AFP levels and show aggressive behavior. In our series, the majority of tumors lacked typical rhabdoid morphology, and all cases of SCUD-HB were found to be INI1 deficient and demonstrated biallelic loss of *SMARCB1* with no other significant copy number alterations or driver mutations identified. In the case series by Vokuhl, more than half of the MRT cases had small cell morphology and were initially classified as SCUD-HB [38]. As our case series and others point out, rhabdoid morphology should not be required in establishing the morphologic diagnosis of MRT. INI-negative SCUD-HB should be recognized as MRT. These tumors have a unique immunophenotype, are usually cytokeratin positive, and are negative for hepatocellular markers (Hep-par1 and Arginase). One potential diagnostic pitfall is that malignant rhabdoid tumors may express Glypican-3 [31] and beta catenin [39], proteins that can also be expressed in hepatoblastomas, therefore these markers are not of use in helping to distinguish between these two different tumor types. In our series, two of six tumors were positive for Glypican-3. 

### 4.3. INI1 Immunohistochemical Staining can Establish the Diagnosis of MRT in the Absence of Rhabdoid Morphology

INI1 immunohistochemical staining can be used to rapidly establish the diagnosis of MRT and avoid delay in obtaining additional molecular studies that establish the diagnosis of MRT. The current clinical treatment of high-risk HB and MRT differs considerably. Historically, many patients with small cell INI1 negative tumors initially received treatment for high risk HB and did not receive MRT-directed treatment until later in their clinical course, after molecular studies established the diagnosis of MRT. 

### 4.4. Elimination of the Term INI Negative HB

Revision of the pathologic classification of liver MRT should eliminate the term INI1 negative SCUD-HB and reclassify as MRT. 

One of the dilemmas faced by pathologists is that, historically, SCUD-HB liver tumors that were confirmed to be INI1-negative were still classified by an international group of pediatric liver pathologists as a subtype of HB, instead of a separate tumor type [24]. The chart from this 2014 publication, listing INI1 negative SCUD-HB, still appears in the nomenclature guidelines published in 2019 by the College of American Pathologists (CAP) [25] and appears in the classification of hepatoblastoma published by the World Health Organization (WHO) in 2019 [40]. Based on our study and recent studies by Vokuhl et al. [38] and Cornet et al. [41], all SCUD-HB with loss of INI1 nuclear staining and small cell morphology are confirmed to have *SMARCB1* deficiencies, supported with analysis utilizing FISH, multiplex ligation dependent probe amplification, and copy number loss of *SMARCB1*. Our study confirmed that SMARCB1 copy number loss is the defining feature of this tumor, with no other significant driver mutations detected utilizing our targeted 467 gene panel (see Supplementary Table S1 for list of genes included in panel). Historically, these cases required additional molecular testing for *SMARCB1* alterations in order to establish the diagnosis of MRT. Given that the majority of MRT cases do not display rhabdoid morphology, or only do in focal areas, particularly with limited sampling of needle core biopsies, there is often a delay in establishing the diagnosis of MRT. 

There has been much confusion in the literature regarding the presence of a focal small cell component in an otherwise typical HB. In the majority of these cases, the focal small cell component is INI1 positive by immunohistochemistry, and none of these INI-positive SCUD-HB tumors have been shown to have *SMARCB1* deletions. Most importantly, the presence of a focal INI-positive small cell component within HB has no clinical implication for aggressive behavior or adverse prognosis [27]. In stark contrast, a small cell liver tumor that is INI1 negative by immunohistochemistry should exclude the diagnosis of HB. One potential diagnostic pitfall, leading to the erroneous diagnosis of a tumor with mixed INI1 negative and INI1 positive components, may result from misinterpreting entrapped hepatocytes as well differentiated fetal components of the tumor [31]. In cases in which immunohistochemical staining results are difficult to interpret, additional molecular studies may be of utility in establishing the diagnosis of MRT. Ancillary molecular studies should not be necessary for establishing the diagnosis of MRT, but are indicated to assess evaluation of the possibility of germline alterations, that may be present in up to 30% of MRT involving other sites. In the vast majority of the cases, INI1 immunostaining is of great diagnostic utility and can rapidly establish the diagnosis of MRT, when integrating this testing with the tumor morphology and immunophenotype in conjunction with other clinical features.

### 4.5. Treatment of Liver MRT

Cis-platinum-based chemotherapy regimens used for HB are ineffective in treating MRT [9]. Previous trials have explored the use of aurora A kinase inhibition in malignant rhabdoid tumors, with an attempt to augment radiation sensitivity in cell lines that overexpress AAK, which occurs in cells with mutations in SMARCB1. However, this treatment was ineffective in producing a response in these tumors [42]. At this time, there are no randomized or prospective trials examining chemotherapy combinations or the addition of new agents, given the rarity of malignant rhabdoid tumor. Only small, retrospective studies from single institutions are present in the literature. Additionally, due to the infrequency of this disease, there is no standard therapeutic pathway. Institutions have treated patients with MRT with commonly used hepatoblastoma therapy, soft tissue sarcoma therapy or a combination of both. Given that MRT is commonly misdiagnosed as a type of undifferentiated hepatoblastoma, standardized treatment regimens have been difficult to establish. Case reports have demonstrated that patients initially diagnosed and treated as hepatoblastomas (with cisplatin-heavy chemotherapy regimens) eventually died of disease progression [41]. 

Reported survivors also seemed to be older (median age 12.5 months)—possibly due to their size and ability to receive higher doses of both chemotherapy and radiation. Treatment with aggressive chemotherapy, in combination with surgery and radiation, has been correlated with improved outcomes, as also demonstrated in two of our patients (3, 6). In another study, 100 patients with a diagnosis of extracranial MRT were registered on the EpSSG Non-Rhabdomyosarcoma Soft Tissue Sarcoma 2005 study (NRSTS 2005) and treated with standard multimodal protocol, including surgery, radiation therapy (RT) and 30 weeks of chemotherapy. Their results showed that age was the only significant factor for overall survival (OS) [43]. A confounding variable in their conclusion was that 14/15 patients who did not receive radiation therapy were <2 years old, as the effects of RT are very difficult to establish in these cases, given the reluctance of physicians to treat young children with radiation therapy. 

Tumors that lack classic rhabdoid morphology have previously been classified as SCUD-HB, even if they show loss of *SMARCB1* or *SMARCA4* by immunohistochemistry (INI1 negative or BRG1 negative, respectively). Many providers will treat a patient diagnosed with SCUD-HB with hepatoblastoma regimens. Chemotherapy regimens for high-risk hepatoblastoma have been established and generally consist of strong alkylators and anthracyclines. A similar treatment approach can be used for hepatic MRT, however these chemotherapy regimens are rarely effective. In our small case series, the two patients that survived underwent several cycles of chemotherapy regimens, borrowed from sarcoma treatment regiments used in treating MRT of other organs. The addition of radiation therapy has also shown some benefit with regards to the outcome. The role is poorly defined, but case reports have demonstrated a possible benefit in cases with microscopic residual disease [44]. Further data must be collected to make a more definitive prediction. Autologous stem cell transplantation has also been used as a means for delivering more myeloablative chemotherapy, however, there is little literature to demonstrate the effectiveness, due to overall small case reports. 

A variety of targeted approaches include the restoration of *SMARCB1* via histone deacetylase inhibitors, however, mice models show these medications to be well-tolerated but with no observable responses [45]. Inhibition of methyltransferase EZH2 has shown durable tumor regression in MRT [46]. The National Cancer Institute Children’s Oncology Group Pediatric Molecular Analysis for Therapy Choice (NCI-COG Pediatric MATCH) trial, launched in 2017, includes treatment with Tazemetostat (EZH2 inhibitor) for tumors with *EZH2, SMARCB1, SMARCA4* mutations/deletions (ClinicalTrials.gov Identifier: NCT03213665).

## 5. Conclusions

In the published literature, most INI1-negative pediatric liver tumors that lack rhabdoid morphologic features have been initially misclassified as SCUD-HB [26,38,41]. In our study, four of six cases were initially diagnosed as SCUD-HB, due to the absence of rhabdoid tumor morphology in the biopsy and/or resection specimen. All six cases showed a loss of INI1 expression by immunohistochemistry and SMARCB1 copy number loss by next generation sequencing, which confirmed the diagnosis of MRT in all six cases. Therefore, based on our findings and data from prior studies [38,41], small cell morphology predominates and rhabdoid morphology is often absent, or a minor component, and is not a robust criterion for diagnosis of MRT. Molecular characterization of INI1 negative tumors has previously been recommended to establish the diagnosis of MRT. In our experience and others [41], the process of characterizing the molecular characteristics of the tumor results in a delay in establishing the diagnosis of MRT. Based on our molecular studies in the appropriate clinical context, liver tumors with small cell or rhabdoid morphology that are both negative for hepatocellular markers (Hep-par1 and Arginase) and INI1-negative are diagnostic of MRT. Further molecular work up should not be required for establishing an MRT diagnosis, but further molecular workup of tumor and non-tumor tissue should be considered in assessing germline changes and excluding rhabdoid tumor predisposition syndrome [33]. In addition, it is critical to eliminate the term INI1-negative HB and classify INI1-negative liver tumors as MRT, since MRT are unique tumors that do not respond to high-risk HB-chemotherapy regimens and may benefit from early targeted therapy.

## Figures and Tables

**Table 1:** Summary of the clinical features and histologic, immunohistochemical and molecular features of tumors.

Case	Age at Diagnosis (Months)	Gender (M/F)	AFP Level (ng/mL)	Specimen Type	Morphology on Biopsy	Initial Diagnosis	GPC-3	Hepatocellular Markers	INI1	CCCP	Subsequent Diagnosis
1	9	M	14.2	B and R	Small cell	SCUD-HB	−	−	Loss	SMARCB1 del	MRT
2	11	F	90	B	Small cell	SCUD-HB	+	−	Loss	SMARCB1 del	MRT
3	3	F	33.8	B and R	Rhabdoid	MRT	−	−	Loss	SMARCB1 del	MRT
4	12	F	69.8	B and R	Small cell *	SCUD-HB	+	−	Loss	SMARCB1 del	MRT
5	10	M	13.6	B	Small cell	SCUD-HB	−	−	Loss	SMARCB1 del	MRT
6	24	M	3.4	B and R	Small cell	MRT	−	−	Loss	SMARCB1 del	MRT

M: male, F: female, B: biopsy, R: resection, AFP: alpha fetoprotein, B: biopsy, R: resection, SCUD-HB: small cell undifferentiated hepatoblastoma, MRT: malignant rhabdoid tumor, GPC-3: glypican-3, Hepatocellular markers: includes Hep-par1 and Arginase immunohistochemistry stains, CCCP: Columbia Combined Cancer Panel, del: deletion; * In Case 4, morphology on resection showed small cell and focal rhabdoid features.

**Table 2:** Summary of tumor treatment and clinical outcome.

Case	Tumor Stage (Evans)	PRETEXT Stage	Preoperative Chemotherapy	Response RECIST	Resection	Site of Metastasis	Radiotherapy	Postoperative/Retrieval Chemotherapy	Outcome
1	I	PRETEXT 2	none	No	R0		Yes Records not available	vincristine, doxorubicin, cyclophosphamide, cisplatin, ifosfamide, etoposide, irinotecan, temozolomide	DOD
2	IV	PRETEXT 3	cisplatin, doxorubicin	No	None	Lung, Brain	No	irinotecan, vincristine	DOD
3	IV	PRETEXT 3M	vincristine, cisplatin, doxorubicin	Yes CR-lung PR- liver	R0	Lung	Yes 1050cGy to whole lung 1050cGy to abdomen	carboplatin, cyclophosphamide, etoposide	Alive at 8 years after diagnosis
4	IV	PRETEXT 2	vincristine, irinotecan, cisplatin, doxorubicin, 5FU	Yes PR- liver PR-lung	R0	Lung	Yes 1050cGy to whole lung 1200cGy to abdomen 14.5Gy to cavity intra-op after recurrence	ifosfamide, carboplatin, vincristine, cyclophosphamide, doxorubicin HD chemotherapy with autologous SCT Second surgical resection	DOD
5	III	PRETEXT 2	cisplatin, doxorubicin	No	None	Lung	No	pazopanib	DOD
6	III	PRETEXT 3	vincristine, doxorubicin, cyclophosphamide	Yes PR-liver	R1 margin		Yes Proton beam 3000cGy to abdomen	HD chemotherapy with autologous SCT	Alive at 1.5 years after diagnosis

DOD: died of disease. Patient 1 had tumor rupture prior to surgery at diagnosis. In Patients 2 and 5, no resection was performed; however, patients received additional chemotherapy (listed under postoperative/retrieval chemotherapy). CR = complete response; PR = partial response.

## Supplementary Materials

The following are available online at https://www.mdpi.com/2072-6694/11/12/1992/s1, Table S1: List of genes included in Columbia Combined Cancer Panel (CCCP) panel.
