# Supplementary material for: Malignant Rhabdoid Tumor, an Aggressive Tumor Often Misclassified as Small Cell Variant of Hepatoblastoma
Source: Cancers (Basel). 2019 Dec 11;11(12):1992. doi: 10.3390/cancers11121992 (PMC6966472; doi:10.3390/cancers11121992)
Supplement: Supplementary file 1 [file cancers-11-01992-s001.pdf]

Malignant Rhabdoid Tumor, an Aggressive Tumor Often Misclassified as Small Cell Variant of Hepatoblastoma

Ladan Fazlollahi, Susan J. Hsiao, Manpreet Kochhar, Mahesh M. Mansukhani, Darrell J. Yamashiro and Helen E. Remotti

Table S1. List of genes included in Columbia Combined Cancer Panel (CCCP) panel.

Columbia Combined Cancer Panel (CCCP) Exons Only												
AKT1	BMPR1A	CDC45	EPHA3	FLCN	IDH1	KMT2C	MRE11A	PDGFRA	PTPRD	SDHB	STAT3	WRN
AKT2	BRCA1	CEBPA	EPHA5	FLT1	IDH2	KMT2D	MSH2	PDPK1	PTPRS	SDHC	STAT5B	WT1
AKT3	BRCA2	CHEK1	EPHB1	FLT3	IFNGR1	KRAS	MSH6	PHF6	PTPRT	SDHD	STAT6	XIAP
ALOX12B	BRCC3	CHEK2	ERBB2	FLT4	IGF1	LAMB4	MTOR	PHF8	PTTG1	SETBP1	STK11	XPA
AMER1	BRIP1	CNOT3	ERBB3	FOXA1	IGF1R	LATS1	MUTYH	PHOX2B	RAC1	SETD2	STK40	XPC
APC	BTK	CREBBP	ERBB4	FOXL2	IGF2	LATS2	MYC	PIGA	RAD21	SF1	SUFU	XPO1
AR	BUB1B	CRKL	ERCC2	FUBP1	IKBKE	LMO1	MYCL	PIK3C2G	RAD50	SF3A1	TP53BP1	YAP1
ARAF	CALR	CRLF2	ERCC3	FYN	IL10	LUC7L2	MYCN	PIK3C3	RAD51	SF3B1	TBL1XR1	YES1
ARID1A	CARD11	CSF1R	ERCC4	GATA1	IL6ST	MAP2K1	MYD88	PIK3CA	RAD51B	SH2B3	TBX3	ZRSR2
ARID1B	CASP8	CSF3R	ERCC5	GATA2	IL7R	MAP2K2	MYOD1	PIK3CB	RAD51C	SH2D1A	TCF3	
ARID2	CBLB	CTCF	ESR1	GATA3	INPP4A	MAP2K4	NBN	PIK3CD	RAD51D	SHQ1	TERT	
ARID5B	CBLC	CTLA4	EXT1	GMNN	INPP4B	MAP3K1	NCOR1	PIK3CG	RAD52	SMAD2	TET1	
ASXL1	CCND3	CUL3	EXT2	GNA11	INSR	MAP3K13	NF1	PIK3R1	RAD54L	SMAD3	TET2	
ASXL2	CCNE1	CYLD	EZH2	GNA13	IRF1	MAPK1	NF2	PIK3R2	RASA1	SMAD4	TET3	
ATM	CD276	DAXX	FAM175A	GNAQ	IRF4	MAX	NFE2L2	PIK3R3	RB1	SMARCA4	TGFBR1	
ATR	CD58	DCUN1D1	FAM46C	GNAS	IRF8	MCL1	NIPBL	PLK2	RBM10	SMARCB1	TGFBR2	
ATRX	CDC6	DDB2	FANCA	GNB1	IRS1	MCM2	NKX2-1	PMAIP1	RECQL4	SMARCD1	TMEM127	
AURKA	CDC7	DDR2	FANCC	GOPC	IRS2	MCM3	NKX3-1	PMS1	REL	SMARCE1	TNFAIP3	
AURKB	CDC73	DICER1	FANCD2	GREM1	JAK1	MCM4	NOTCH1	PMS2	RFWD2	SMC1A	TNFRSF14	
AXIN1	CDCA5	DIS3	FANCE	GRID1	JAK3	MCM5	NOTCH2	PNRC1	RHOA	SMC3	TOPBP1	
AXIN2	CDH1	DNM2	FANCF	GRIN2A	JUN	MCM6	NOTCH3	POLE	RICTOR	SMO	TP53	
AXL	CDK12	DNMT1	FANCG	GSK3B	KCNJ5	MCM7	NOTCH4	POT1	RIT1	SOCS1	TP63	
B2M	CDK4	DNMT3A	FAS	H3F3A	KDM5C	MDC1	NRAS	PPP2R1A	RNF43	SOX17	TRAF7	
BAP1	CDK8	DNMT3B	FAT1	H3F3C	KDM6A	MDM2	NT5C2	PRDM1	RPL10	SOX2	TSC1	
BARD1	CDKN1A	DOT1L	FBXO11	HGF	KDM6B	MDM4	NTRK2	PRF1	RPL5	SOX9	TSC2	
BBC3	CDKN1B	E2F3	FBXW7	HIST1H1C	KDR	MED12	PAK1	PRPF40B	RPS6KA4	SPEN	TSHR	
BCL11B	CDKN2A	ECT2L	FGF19	HIST1H2BD	KEAP1	MEF2B	PAK7	PRPF8	RPS6KB2	SPOP	U2AF1	
BCL2L1	CDKN2B	EED	FGF3	HIST1H3B	KIAA1549	MEN1	PALB2	PTCH1	RPTOR	SRC	U2AF2	
BCL2L11	CDKN2C	EGFL7	FGF4	HNF1A	KIT	MET	PARK2	PTEN	RYBP	SRSF2	UBR5	
BCL6	CDT1	EGFR	FGFR2	HRAS	KLF4	MITF	PARP1	PTPN1	SBDS	STAG1	VHL	
BCORL1	CD79A	EIF1AX	FGFR4	ICOSLG	KLF6	MLH1	PBRM1	PTPN11	SDHA	STAG2	VTCN1	
BLM	CD79B	EPCAM	FH	ID3	KLHL6	MPL	PDCD1	PTPRC	SDHAF2	STAG3	WAS	
**Columbia Combined Cancer Panel (CCCP) Whole Genes**												
ABL1	BRD4	CIITA	EP300	EWSR1	FUS	KAT6A	NPM1	PAX5	PPARG	RUNX1	SUZ12	TPM3
ALK	CBL	CLTC	ERG	EZR	GPC3	KIF5B	NTRK1	PAX8	PRKAR1A	SLC45A3	SYK	USP6
BCOR	CD274	CLTCL1	ETV1	FGFR1	HMGA2	KMT2A	NTRK3	PDGFRB	RAF1	SS18	TAF15	
BCR	CD74	CREBBP	ETV4	FGFR3	IL2	LRIG3	NUP214	PICALM	RARA	SSX1	TCF12	
BRAF	CDK6	CRLF2	ETV5	FIP1L1	ITK	MECOM	NUP98	PLAG1	RET	SSX2	TFE3	
BRD3	CIC	CTNNB1	ETV6	FOXO1	JAK2	MLLT10	NUTM1	PML	ROS1	SSX4	TMPRSS2	

© 2019 by the authors. Licensee MDPI, Basel, Switzerland. This article is an open access article distributed under the terms and conditions of the Creative Commons Attribution (CC BY) license (http://creativecommons.org/licenses/by/4.0/).
